# Adaptive radiotherapy based on statistical process control for oropharyngeal cancer

**DOI:** 10.1002/acm2.12993

**Published:** 2020-08-08

**Authors:** Hesheng Wang, Jinyu Xue, Ting Chen, Tanxia Qu, David Barbee, Moses Tam, Kenneth Hu

**Affiliations:** ^1^ Department of Radiation Oncology NYU Langone Health New York NY USA

**Keywords:** adaptive radiotherapy, head and neck cancer, oropharyngeal cancer, statistical process control

## Abstract

**Purpose:**

The purpose of this study is to quantify dosimetric changes throughout the delivery of oropharyngeal cancer treatment and to investigate the application of statistical process control (SPC) for the management of significant deviations during the course of radiotherapy.

**Methods:**

Thirteen oropharyngeal cancer patients with daily cone beam computed tomography (CBCT) were retrospectively reviewed. Cone beam computed tomography images of every other fraction were imported to the Velocity software and registered to planning CT using the 6 DOF (degrees of freedom) couch shifts generated during patient setup. Using Velocity “Adaptive Monitoring” module, the setup‐corrected CBCT was matched to planning CT using a deformable registration. Volumes and dose metrics at each fraction were calculated and rated with plan values to evaluate interfractional dosimetric variations using a SPC framework. *T*‐tests between plan and fraction volumes were performed to find statistically insignificant fractions. Average upper and lower process capacity limits (UCL, LCL) of each dose metric were derived from these fractions using conventional SPC guidelines.

**Results:**

Gross tumor volume (GTV) and organ at risk (OAR) volumes in the first 13 fractions had no significant changes from the pretreatment planning CT. The GTV and the parotid glands subsequently decreased by 10% at the completion of treatment. There were 3–4% increases in parotid mean doses, but no significant differences in dose metrics of GTV and other OARs. The changes were organ and patient dependent. Control charts for various dose metrics were generated to assess the metrics at each fraction for individual patient.

**Conclusions:**

Daily CBCT could be used to monitor dosimetric variations of targets and OARs resulting from volume changes and tissue deformation in oropharyngeal cancer radiotherapy. Treatment review with the guidance of a SPC tool allows for an objective and consistent clinical decision to apply adaptive radiotherapy.

## 1 INTRODUCTION

Conventional dose regimens with intensity‐modulated radiation therapy (IMRT) or volumetric modulated arc therapy (VMAT) have been considered a standard treatment of head and neck (HN) cancers. During the long course of treatment over 30 plus days, however, patients often experience the changes of anatomy in both the shape and the volume. The anatomic changes would evidently result in the deviation of the delivered dose from the planned. The consequence could be potentially underdosing to the treatment target volumes and overdosing to the organs at risk (OARs).^1^, ^2^

The deviation of the delivered dose has long been a clinical concern since it can increase the risk of normal tissue complications and decease the probability of tumor control.^3^ It is suggested that anatomic changes be monitored during the course of treatment and the treatment plan be modified as necessary to correct the dose.^4^, ^5^, ^6^ Cone beam computed tomography (CBCT) has been used to align the target in image‐guided radiotherapy (IGRT) process. Cone beam computed tomography is also useful to visualize the anatomic changes. Adaptive radiotherapy (ART) is therefore proposed as the treatment process that employs online imaging on a routine basis to monitor the anatomic changes and to determine when it is necessary for replanning.^7^, ^8^, ^9^

Clinical implementation of ART is a challenge in several aspects. Clinicians need to understand what patients would benefit from ART, when replanning is considered necessary and how often it should be performed. In addition, evaluation of dosimetric deviations associated with the anatomic changes is not trivial. Currently, setup CBCT is often used to coregister with the planning CT and dose is deformed to the CBCT. Many studies have been published, which investigated the anatomic changes and the dosimetric changes during radiotherapy of HN cancers, and a few of which also discussed the implications of those changes on tumor control and normal tissue complications. The review by Brouwer CL^10^ found no consensus or a clear guideline about the criteria for the application of ART in clinic.

This study investigated both anatomic and dosimetric changed for each fraction of HN cancer treatment and attempted to establish a tangible criteria for the decision making as to when treatment should be intervened for replanning.

## 2 MATERIALS AND METHODS

### 2.A Radiation treatment and CBCT

This IRB‐approved study (#I18‐00659) retrospectively analyzed 13 patients (11 male and 2 female) who received radiotherapy for oropharyngeal cancer. The patients were with a median age of 62 yr old (range: 52–86 yr old). The patients were treated with either 11‐field IMRT or 2–3 arc VMAT in 35 or 33 fractions for 7–8 weeks (5 fractions/week). Target volume for gross tumor volumes (GTVs) was prescribed to 70 or 66 Gy (200 cGy/ fraction), respectively. Fractional doses of 190, 180, and/or 165 cGy were also prescribed for additional target volumes of various risks containing microscopic diseases. All plans were generated from treatment planning CT acquired on a SOMATOM Definition AS CT scan (Siemens, Munich, Germany) typically with 205 mA, 120 kVp, 1.3 × 1.3 mm axial resolution and 3‐mm‐slice thickness. Varian Eclipse treatment planning system (TPS) (Eclipse 13.7, Varian Medical System, Inc.) was used for planning and optimization.

All the patients were immobilized with thermoplastic head masks and treated on Varian TrueBeam machines with image guidance of the on‐board kV‐CBCT. CBCT images were acquired at each fraction prior to irradiation using a standard Head CBCT mode with parameters of 100 kV, 15 mA, Full fan. and half trajectory. CBCT volume (resolution: 0.05 × 0.05 × 0.2 cm) covers anatomy from brainstem to neck with a scanning length of approximate 19 cm. The daily CBCT is subsequently registered to planning CT for patient setup with 6 DOF couch shifts. The registration is focused on alignment of GTV/CTV target, and reviewed/approved by physicians following a standard verification protocol which includes on‐line review prior to delivery of first fraction and off‐line review before procedure of following treatment fractions. In addition, physicists also review the registrations on a weekly base and communicate with therapists and physicians for any concerns on the setup errors.

### 2.B Registration procedure

Patient planning CT, dose volume, structure set, and CBCT of every other fraction were imported into Velocity (Velocity 4.0, Varian Medical System, Inc.) for analysis. For preprocessing, we applied the pretreatment rigid transformations obtained at console during patient setup to respective CBCT to remove the impact of residual patient setup errors. Once registered, the dose volume defined on the planning CT was projected to the CBCT domain as both share the same isocenter and coordinate system. Subsequently, velocity “adaptive monitoring navigator” (AMN) module was used to monitor the volumetric and dosimetric variations during the treatment course for selected set of structures (GTV/CTV/selected OARs). For the first step of the AMN deformable registration from CBCT (secondary volume) to the corresponding planning CT (primary volume) was performed for each CBCT (total 216 registrations for 13 patients) with the volume of interest (VOI) set as the valid CBCT volume minus two slices from top and bottom to alleviate the uncertainty of registration at the image boundaries. Registration results were visually assessed by experienced physicists for the anatomical conformity of deformed CBCT with planning CT and the physical plausibility of underlying deformation vector maps. For volumetric and dosimetric evaluation, we cropped target volumes and OARs within the registration VOI. Then we used the CBCT to CT registration inversely to deform selected structures in CT into CBCT images. The volumes for these structures at each fraction were calculated using the deformed structures, and adaptive DVHs at subsequent treatment fractions were also calculated using the deformed structures in CBCT and planned dose projected to CBCT, under the assumption that daily volumetric dose distribution had not significant change.^10^ Thereby the change in volumes and DVH metrics for selected structures during the treatment course were reconstructed for statistical analysis.

### 2.C Statistical process control (SPC)

*T*‐tests between target volumes (GTV) in CT plans and fractional CBCTs showed no significant volume changes until the 13th fractions (or 17 days from first treatment) on the whole cohort of patients under study. Using the data from the first 17 days, we calculated average and standard deviation (STD) of each dose metric, and created control chart for the metric with upper control limit average variations of volume (UCL: average + 3 × STD) and lower limit (LCL: average‐3 × STD). Upper control limit and LCL can be specific on patient cohort used, clinical procedure followed, and structures of concern applied. Table 2 shows the mean, UCL and LCL of target and OAR dose metrics based on our patient cohort of this study.

## 3 RESULTS

The change in the shape and volume of each structure was observed from the translational deviation of the center of a structure over the course of treatment. Summarized in Table 1 are the shifts in the three Cartesian directions for targets and some OARs. The structures had absolute shifts in L–R and anterior–posterior (A–P) directions as much as 1.0, and 1.5 cm in S–I direction. A consistent 1‐mm shift present for the structures in the A–P direction, suggesting systematic setup displacement in A–P direction.

**Table 1:** Variations of anatomic changes in both the shape and volume of each structure by the translational deviation of the structure's center along the left/right (L/R), anterior/posterior (A/P) and superior/inferior (S/I) direction, respectively.

	L/R shift (mm)		A/P shift (mm)		S/I shift (mm)	
	Median	Max	Median	Max	Median	Max
GTV	1.4	9.4	1.6	5.8	1.4	14.8
CTV	1.4	8.5	1.6	5.6	1.3	14.4
Lt parotid	1.3	8.3	1.7	6.2	1.4	9.9
Rt parotid	1.2	7.3	1.7	7.9	1.6	10.1
Oral cavity	0.8	6.4	1.5	8.5	1.8	11.3

**Fig. 1:**
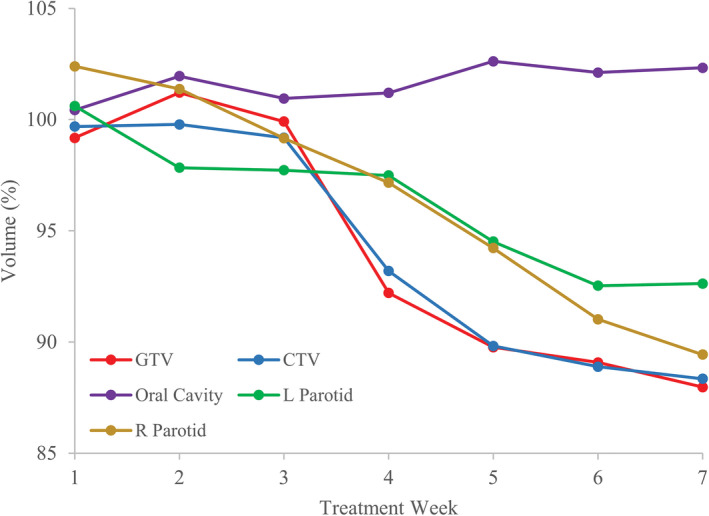
Percentage of median volume at 1st fraction of each week in the treatment course with respect to the volume from the planning computed tomography.

Figure 1 plots the volumes of targets and some OARs in percentage of the mean in the cohort at the first fraction of each week relative to the planning CT volume through the treatment course. In general, the volumes of the structures had minor changes in the first 2 weeks of the treatment, subsequently, the volume shrunk down over time for most structures although the amount is <15% at the 7th week. In comparison, oral cavity present insignificant variations of weekly changes in volume, consistent with the fact that the oral cavity is a rigid structure.

**Fig. 2:**
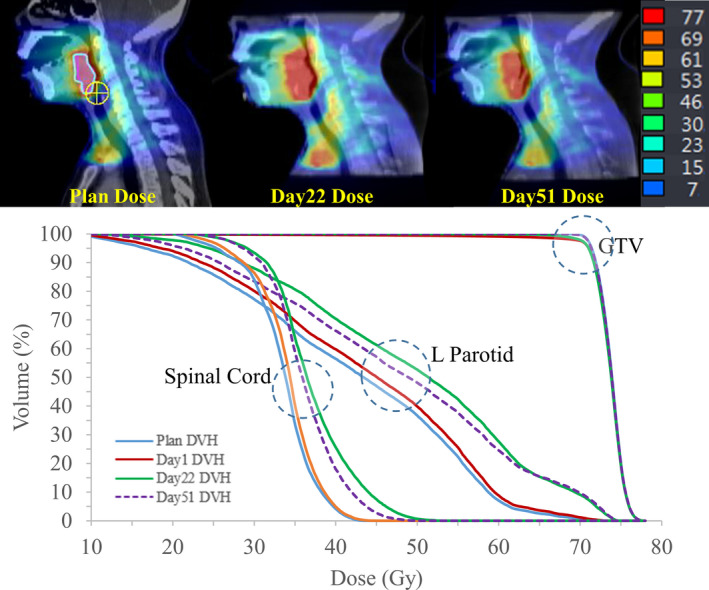
Dose distribution and DVHs at plan and different fraction days.

Presented in Fig. 2 is an example case of dose distribution on planning CT as well as coregistered CBCTs on different days of treatment. DVHs for GTV and OARs on different days of treatment are also shown to be compared with the planned. DVHs of the left parotid and spinal cord had substantial elevations in late treatment fractions compared with the planned values, whereas GTV coverage appears fairly constant.

**Fig. 3:**
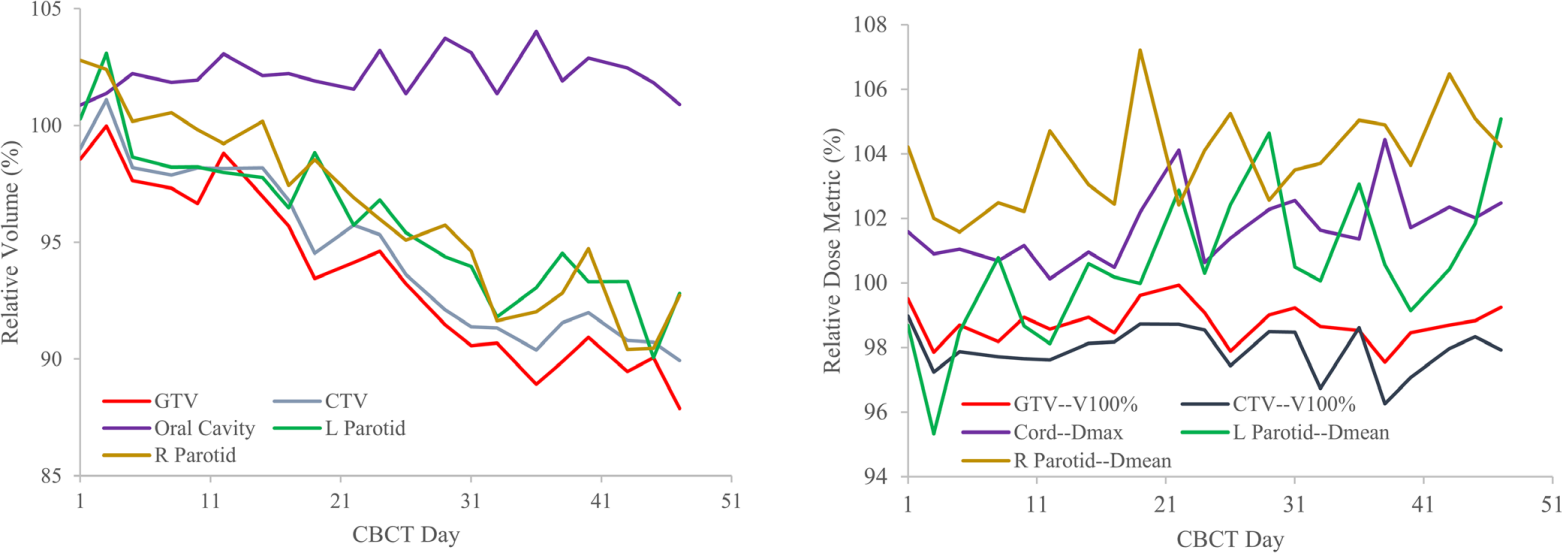
Variation of average volume (left) and dose metrics (right) relative to respective plan values throughout the treatment course.

Figure 3 shows the variations of average volume and dose metrics on the daily basis which were calculated by a deformed registration with the CBCT from the specified day. Gross tumor volume and parotids had ~10% shrinkage in volume at the end of treatment. Oral cavity volume, which is not dependent on the radiation received, is also plotted, showing the robustness of the volume monitoring. Noticeably, daily dosimetric changes can be fluctuating throughout the course of treatment whereas changes in accumulative dose metrics are more clinically relevant. The change in parotid mean dose is seen <5%.

**Fig. 4:**
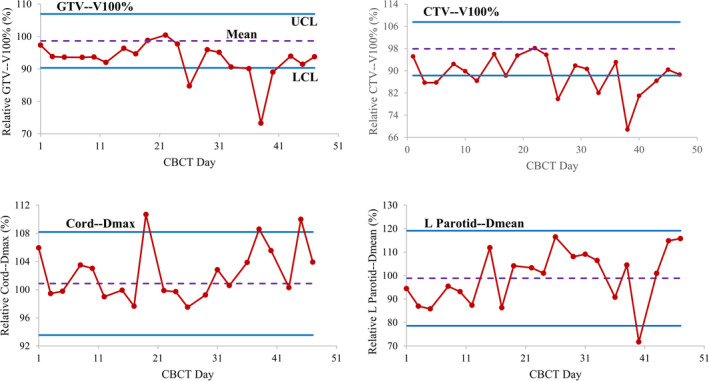
Control charts assessing dose changes of individual patient during treatment.

Table 2 presents mean, UCL and LCL for dose metrics of target and OARs calculated from the first 13th fractions from our own patient cohort. Using the measurements, Fig. 4 evaluates four dose metrics changes for a patient by control charts. Dose metric is seen fluctuating over the course of treatment and out of the limit for a few continuing number of fractions. The former might be related to daily setup variations and the latter was likely a result of the anatomic changes. Each dose metric of concern has its own control chart. The control chart can be utilized individually or collectively to assess dosimetric changes based on physician’s priorities on dose constraints of each structure.

**Table 2:** Mean, upper and lower process capacity limits (UCL and LCL) of target and organ at risk (OAR) dose metrics.

	GTV–V100%	CTV–V100%	Cord–Dmax	L parotid–Dmean	R parotid–Dmean
Mean (%)	98.6	97.9	100.9	98.9	102.8
UCL (%)	106.9	107.6	108.2	119.1	123.7
LCL (%)	90.3	88.3	93.6	78.6	82.0

V100%: percentage volume receiving 100% prescription dose; Dmax: max dose; Dmean: mean dose.

## 4 DISCUSSIONS

Anatomic changes are expected on a daily basis. The change in both the shape and the volume of each structure was observed for every patient in this study, especially at the late weeks of the long treatment course. The review by Brouwer et al.^11^ previously presented information about the possible cause and the variation of anatomic changes in a number of OARs as well as the associated dosimetric changes as available. As reported by most studies,^12^ ipsilateral parotid glands tend to shrink and to shift medially toward the high dose region over the course of treatment, potentially jeopardizing parotid sparing. The effect of anatomic changes on dose is complex and patient dependent. There is no direct correlation or descriptive models between anatomic and dosimetric changes for a particular OAR being established.

In agreement with other studies,^13^, ^14^, ^15^, ^16^ dose to clinical target volume (CTV) is less sensitive in this study than dose to most of OARs simply due to the planning target volume (PTV) margin being applied to the target. In general, the magnitude of dose deviations is larger for OARs than targets if no planning margin is employed for an OAR. Planning volume at risk (PRV) has been used by some clinicians for spinal cord and brainstem, but it is not common for all OARs involved in the treatment of head and neck cancer.^11^

The clinical concern is lower tumor control and higher normal tissue complications associated with anatomic changes. Very few studies presented the quantitative correlation of the changes in tumor control or normal tissue complications with anatomic changes, and only limited data were available about how dosimetric changes would affect the treatment outcomes.^11^ It is demonstrated^17^, ^18^ that reoptimization of plans to the changes of anatomy did improve the dose distribution of HN cancer patients. It remains an unanswered question as to what extent the dosimetric changes would result in unacceptable or suboptimal clinical outcomes. Necessity for an intervention with adaptive therapy should ultimately be based on the clinical significance of the deviations in either tumor control or normal tissue complications anticipated.

Decision for replanning has been highly subjective to the attending radiation oncologists, who often decide by comparing anatomic changes in the setup CBCTs with the planning CT. There are no established criteria, which correlates the clinical decision making for resimulation and replanning with the outcomes. Recent study by Zhang et al.^19^ retrospectively investigated some patients in their clinic with and without an intervention of ART. Greater than 5% difference in the dose to the CTV or <0.75 Pearson correlation coefficient of the CTV was found to be the action level related to the clinical decision made for ART.^20^ This study has made one step forward for developing the clinically relevant criteria, although the study does not have meaningful outcomes data to support their action levels. In addition, their decision‐making criteria for ART were solely based on the dosimetric or geometric deviations of the target, which is normally less of an issue than those of the OARs.^11^

Our study using statistical process control is the approach to the clinically tangible criteria that would allow for the decision‐making based on the anticipated outcomes of concern. Thresholds of both high limit (HL) and low limit (LL) are generated for each dose metric of targets and OARs of a patient. Furthermore, a global HL and LL could be established by building SPC in a patient cohort. Optimal UCL and LCL should be developed with more patients and best be correlated with the clinical evidence. The SPC could provide a guidance for the action level when resimulation and replanning is expected.

As the start point to implement the procedure, clinicians can test their own patient cohort. Dose metrics for the structures of concern should be monitored to understand any trending. A clinical procedure can be worked out with the data and experience they have obtained. The same statistical *t*‐test can be applied to determine the time point of the fraction number at which significant changes in the target volume occur. Any dose metric out of the bounds is an alert for review and quality control. Based on clinical priorities for dosimetric constraints of the target volumes and critical structures, immediate attention may be needed when a fraction has dose metrics out of the limits. Further monitoring for the trend in the SPC or resimulation for ART could be determined. The action levels for an ART intervention can be established by statistical, biological, or clinical significance.^19^ The control chart provides a statistical yet objective baseline for clinicians to consider any necessary interventions along the course of treatment. This method can ultimately be refined to associate the action levels in dosimetric changes with the clinical outcomes.

The majority of this study involved deformable registration of daily CBCT with planning CT, from which the volume of structures on the treatment day was derived. Accuracy of the registration varies with the degree of anatomical changes and the quality of CBCT. The commercial deformable image registration in Velocity has been assessed by various studies using virtual phantoms, thorax phantom, and patient data.^20^, ^21^ We also visually checked anatomical alignments from the registrations for at least one CBCT in a week treatment delivery. Nevertheless, separate quality assurance of the CBCT and CT deformable registration is necessary and an automated method may aid in the procedure.^22^

In the current workflow of AMN, the change of dose distribution in each fraction is ignored when DVHs are reconstructed for evaluation. The assumption that the dose volume did not change with respect to the treatment plan during the course of treatment played a critical role in the reconstruction of DVHs of structures at treatment. However, the assumption itself is not always valid throughout the treatment course. As the patient anatomy changes, the external body surface, as well as the internal relative location of organs, deviated away from the treatment plan. This interfractional change of the patient anatomy altered the HU/density distribution in the spatial domain of the treatment plan. Therefore the actual fractional dose distribution, which was calculated based on the underlying anatomy, could be different from the planned dose volume. The impact of the change of dose distribution can be significant. To improve the accuracy of the adaptive DVHs, dose volume needs to be recalculated using the anatomy information in the CBCT. One of the problems preventing us from performing dose recalculation directly on CBCTs was the lack of CBCT HU/density calibration curve. Future work is underway to implement CBCT‐based dose recalculation using CBCT to synthetic CT conversion^23^ or CBCT HU override.^24^

## 5 CONCLUSIONS

Daily CBCT could be used to monitor dosimetric changes in both targets and OARs due to the volumetric changes and organ deformation during the course of oropharyngeal HN cancer radiotherapy. We developed a method with statistical process control, which can be used to establish the clinical criteria for ART by analyzing the correlation of dosimetric changes with the outcomes data. Treatment review with guidance of an SPC tool enable radiation oncologists to objectively and consistently identify the fractions with dosimetric changes that are of clinical significance.

## CONFLICT OF INTEREST

None of the authors has conflict of interest or funding to disclose related to the work of this publication.
